# Identification of gene–sun exposure interactions of GWAS-identified variants in perceived facial aging progression

**DOI:** 10.3389/fragi.2025.1519799

**Published:** 2025-07-23

**Authors:** Ludivine Obry, Raissa Medina-Santos, Myriam Rahmouni, Josselin Noirel, Toufik Labib, Pilar Galan, Jean-Louis Spadoni, Gaëlle Gendronneau, Randa Jdid, Sandra Courrèges, Julie Latreille, Nada André, Jean-François Zagury, Sigrid Le Clerc

**Affiliations:** ^1^ Laboratoire Génomique, Bioinformatique, et Chimie Moléculaire, EA7528, Conservatoire National des Arts et Métiers, Paris, France; ^2^ Equipe de Recherche en Epidémiologie Nutritionnelle (EREN), Centre d'Epidemiologie et Biostatistiques Sorbonne Paris Cité (CRESS), Inserm U1153, Inra U1125, Cnam, Université Sorbonne Paris Nord et Sorbonne-Paris-Cité, Paris, France; ^3^ Biological and Clinical Department, IRD Chanel Fragrance & Beauty, Bobigny, France

**Keywords:** G×E interaction, perceived age, facial aging, SNP, sun exposure, GWAS

## Abstract

**Background:**

Skin aging is characterized by observable major changes in the composition and organization of the skin, including the appearance of wrinkles, tissue sagging, dryness, and pigmentary disorders. While both environmental and genetic factors contribute to these changes, their interaction remains underexplored. Perceived age is a biomarker of health and strongly related to facial skin aging features. Thus, we conducted a gene-environment interaction analysis on the perceived facial aging progression phenotype in 226 women, focusing on sun exposure as the environmental factor.

**Methods:**

We assessed perceived age in 226 women at two time points, 12 years apart, allowing defining a perceived facial aging progression as phenotype. We applied a two-step gene-environment interaction approach. First, a genome-wide association study (GWAS) was performed to identify SNP effects on the perceived facial aging progression, selecting those with a *p*-value <5 × 10^−3^. In the second step, we used GEM software to test interactions between the 7,464 selected SNP and sun exposure.

**Result:**

The GWAS identified four significant SNP associated with perceived facial aging progression, located in the *CGGBP1*, *PGM5-AS1*, and *CSMD1* genes. The *CGGBP1* gene is involved in DNA damage/repair, telomere and mRNA metabolism; *PGM5-AS1* is an antisense RNA regulating *PGM5*, a component of adherens-type cell junctions; *CSMD1* is involved in complement regulation and cell migration. In the second step, we tested 7,464 SNP for interactions with sun exposure in perceived facial aging progression, identifying a significant interaction signal in the *VANGL1* gene. The *VANGL1* gene contributes to the structural organization of the plasma membrane and has been identified as a key gene for cutaneous function and was expressed during keratinization.

**Discussion:**

This study revealed four relevant genetic associations with skin aging and one significant G × E interaction. These findings support the known link between telomere shortening/protection and aging, and suggesting a potential role for keratinization in the context of facial aging and sun exposure, though further validation in larger cohorts is necessary. The findings could help to develop new approaches for facial aging prevention and treatment and to better understand molecular mechanisms of aging.

## Introduction

Skin aging is characterized by observable major changes in the composition and organization of the skin, including the appearance of wrinkles, tissue sagging, dryness, and pigmentary disorders. These changes are also accompanied by alterations in the underlying skeletal and adipose structures of the face, which further contribute to the overall appearance of aging (Coleman and Rajiv, 2006; Ng and Chew, 2022). Both environmental and genetic factors significantly influence facial aging, and their respective contributions have been studied (Ng and Chew, 2022; Krutmann et al., 2017). While these factors have been studied individually, less is known about how they interact and contribute to the aging process collectively (Krutmann et al., 2017).

The interaction between genetic variations and environmental factors (G × E) have a significant impact on complex human traits and diseases, including skin and facial aging (Krutmann et al., 2017; Herrera-Luis et al., 2024). The skin serves as an excellent model for studying G × E interactions, as it undergoes both intrinsic aging and extrinsic aging in areas exposed to environmental factors, such as the face (Krutmann et al., 2021). Genetic predispositions may influence how skin responds to environmental stressors such as UV radiation and pollution, thus investigating these interactions could provide critical insights into the underlying biological processes of skin aging and overall progression of the aging process (Krutmann et al., 2021).

Among the high number of skin aging phenotype definitions, perceived facial age, a subjective assessment based on an individual’s appearance, stands out as a relevant phenotype for studying G × E interactions due to its inclusion of several features related to skin aging and its association with both genetic and environmental factors (Ng and Chew, 2022; Roberts et al., 2020; Avila et al., 2023; Voegeli et al., 2021). While perceived facial aging is mainly explained by skin aging features (wrinkles, sagging and hyperpigmentation), the muscles, the fat distribution, and the ligaments contribute to the complex underlying anatomy of the face that modulates perception of facial aging (Gunn et al., 2009; Nkengne et al., 2008; Merinville et al., 2015; Swift et al., 2021; Cotofana et al., 2016). Consequently, Perceived age is multifactorial process, not limited to skin changes. Moreover, perceived facial aging is a robust biomarker of ageing that predicts survival in elderly individuals and health (Christensen et al., 2009; Rippon and Steptoe, 2015). It therefore provides a holistic phenotype for G × E studies on facial aging and overall health.

To further explore the role of G × E interactions in perceived facial aging, we assessed perceived age in 226 women at two time points, 12 years apart, allowing defining a perceived facial aging progression (PFAP) as phenotype. Then, we conducted a two-step study focusing on perceived age and sun exposure, allowing reducing the multiple testing burden by focusing on SNP likely to be involved in interactions (Gauderman et al., 2017). In the first step, a genome-wide association study (GWAS) was performed to identify SNP with potential marginal genetic effect on PFAP, and in the second step, we tested these SNP for interaction with sun exposure.

## Materials and methods

### Cohort

A cross-sectional study was conducted to investigate skin aging in the context of the SU.VI.MAX cohort, a longitudinal cohort study, conducted in French middle-aged adults (Galan et al., 1998). The protocol was approved by the Hospital Medicals Ethics Committee of Paris-Cochin (CCPPRB no. 706) and the ‘‘Commission Nationale de l’Informatique et des Libertés’’ (CNIL no. 334641). The study was conducted according to the Declaration of Helsinki Principles. All participants gave their written, informed consent. The SU.VI.MAX cohort included 13,017 volunteers who were representative of the French adult middle-aged population for most sociodemographic features (Hercberg et al., 2004). The individuals were extracted from SU.VI.MAX in 2009 to investigate skin aging genes in women. 570 women, aged 44–70 years, took part in this study and provided their informed consent (Le Clerc et al., 2013).

### Assessment of perceived age and sun exposure

Among the 570 women who took part in this study in 2002, 68 were excluded due to quality control (see details in Le Clerc et al., 2013). Among the remaining 502 women, 226 participated in the second round of phenotyping in 2014 and were available for the analysis of perceived facial aging progression.

### Lifetime sun exposure

Lifetime sun exposure intensity was estimated in 2002 by a score based on data collected by a self-reported questionnaire. This score is a linear combination of five items weighted according to their relative contribution to the score: voluntary sun exposure, exposure of the body and the facial skin, exposure during the hottest hours of the day, intensity of self-reported lifetime sun exposure, and consideration for sunbathing. The design, validation, and description of this score have been described previously (Guinot, Malvy, and Latreille, 2001). This score was categorized into seven categories, with values ranging from 1 (lowest) to 7 (highest) exposure. The unavoidable potential bias linked to self-reported questionnaire is one limitation of our study.

### Perceived age

Facial photographs were collected in 2002 and 2014 for 226 women. Three standardized, high-resolution digital images of the face were taken for each participant (one frontal view of the face and one of each profile) using a Kodak digital camera (Kodak, Paris, France) (Supplementary Figure S1). The camera was mounted on a monopod and a specifically developed chair was used to allow standardized positions of the camera with respect to the face. Lighting conditions were standardized by means of two symmetrical lamps, which provided a continuous daylight spectrum, placed at 45° to each side of the face. The participants were asked to follow specific skin care instructions; notably, application of detergents or cosmetics to the face was not authorized for at least 12 h before the study visit. They were also instructed to avoid applying makeup to their facial skin and wearing jewelry in the morning of the day of imaging. Hair was masked using a white headband. A high-resolution digital image of the frontal view of the face, with a neutral facial expression, and eyes closed was taken.

The assessment of perceived facial age involved 60 French naïve judge Caucasian women aged 30 to 70, recruited via a professional agency that provided compensation. Only women were included, as they are generally more accurate in estimating other women’s ages in everyday contexts. Inclusion criteria required participants to be French-speaking women within the target age range. Exclusion criteria included vision problems and insufficient understanding of French. Photographs were sequentially and randomly presented to the graders on a computer screen. They were requested to give subject age using E-Prime software interface that featured a slider bar spanning from 0 to 100 for age estimation (Taylor and Marsh, 2017). Photos were grouped into two blocks by year (2002 and 2014), with half the participants starting with one block or the other. To manage cognitive load, an incomplete block design was used: each participant rated 150 randomly selected photos per block. Each facial photograph was estimated by at least 33 judges (from 33 to 42 judgments). The average perceived facial age was used for the analysis.

In this study, we aimed to study a perceived facial aging progression rather than a state. For this purpose, we defined the perceived facial aging progression as:

PFAP=perceived facial age in 2014−perceived facial age in 2002chronological age in 2002

### Genotyping/imputation

The 226 women were genotyped using Illumina Infinium HumanOmni1-Quad BeadChips (Illumina, San Diego, CA) that contain 1,140,419 markers. After quality control, a total of 795,063 SNP remained for imputation. To correct for possible population stratification, genotypes were analyzed using EIGENSTRAT utility of the EIGENSOFT package version 8.0 (Price et al., 2006) to perform a principal component analysis (PCA). As a result, the 226 individuals exhibited a European ancestry and the two first component of PCA were used in the statistical analyses to avoid geographical stratification bias (Supplementary Figure S2). The genotype data were phased using SHAPEIT4 (Delaneau et al., 2019). The phased data were then imputed using IMPUTE2 (Howie et al., 2012). As reference haplotypes, we used genotype data of 2,054 individuals from the phase 3 integrated variant set of the 1000 Genomes project released in 2014 (1000 Project Consortium et al., 2010). After imputation and quality control (MAF >5%, imputation *r* ^2^ > 0.3) we obtained 6,897,523 SNP for 226 individuals.

### Statistical analyses

We performed a two-step procedure for analysis of G × E interaction, allowing the reduction of the multiple testing burden by focusing on SNP more likely to be involved in interactions (Gauderman et al., 2017). During the first step, we conducted a classical GWAS to assess the marginal genetic effects regarding the PFAP. We used SNPTEST software to perform linear regression with an additive model between genotype and PFAP, and we added the two first principal component of the PCA and the categorized score sun exposure as covariates (see Lifetime sun exposure), (Marchini et al., 2007). We used the genome-wide threshold of 5 × 10^−8^ to define significant SNP of the GWAS. For subset testing in a 2-step procedure, we considered the GWAS *p*-value threshold of 1 × 10^−3^. The second step consisted of testing the G × E interaction for perceived age phenotype and was done using GEM software with a linear regression model and with the two first principal component of the PCA as covariates and the categorized sun exposure score as interaction variable (Westerman et al., 2021). We obtained a *p*-value for the interaction effect and one for the genetic and interaction effects together, namely, the joint *p*-value. We tested the G × E interaction based on interaction effect alone between selected SNP and sun exposure regarding the PFAP phenotype. We computed linkage disequilibrium (LD) with LDlink between the selected SNPs in the GWAS, to define a set of independent SNP (LD *r* ^2^ < 0.8) and the Bonferroni threshold for the interaction analysis (0.05/number of independent SNP).

### Bioinformatics investigations

Following the association and interaction analyses, we carried out investigations to explore the potential functional role of the SNPs in gene expression regulation. First, we used the tool RegulomeDB (Boyle et al., 2012) and the scoring scheme of this tool refers to a higher likelihood of being functional (with 1 being higher and 7 being lower score). Then we explored several databases related to quantitative trait loci: eQTL in GTEX (Sobin et al., 2024); mQTL in mQTLdb and in EPIC DB (Gaunt et al., 2016; Villicaña et al., 2023); pQTL in SCALOP (Folkersen et al., 2020). A mQTL was considered if it existed in both mQTLdb and EPIC DB. The linkage disequilibrium was assessed using LDlink in the CEU population of 1000 genomes (Machiela and Chanock, 2015).

## Results

To explore the interaction between genetic variations and sun exposure in perceived facial aging, we conducted a two-step study, using an initial screening to prioritize SNP that are more likely to be involved in an interaction and testing for interaction in a second step (Gauderman et al., 2017). We assessed perceived age in 226 women at two time points, 12 years apart, allowing us to define a perceived facial aging progression (PFAP) phenotype used as facial and skin aging phenotypes.

**FIGURE 1:**
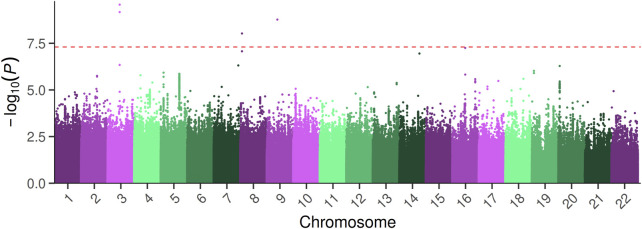
Manhattan plot of the GWAS analysis on PFAP. The–log_10_ (*p*-values) of the GWAS are plotted across chromosomes along the x-axis. The red dashed line indicates the genome-wide significance threshold (*p*-value = 5 × 10^−8^).

In the first step, we performed a classical GWAS focused on PFAP on 6,897,523 SNP (MAF >5%; imputation *r* ^2^ > 0.3). We identified four significant SNP (rs114462394, rs77074982, rs567952380, rs75470026) associated with PFAP (Table 1; Figure 1). The four SNP showed imputation *r* ^2^ between 0.3 and 0.4, suggesting a high level of uncertainty in the imputed genotypes. However, the MAF of these SNP in our sample were similar to those in the 1000 Genomes European population (Table 1). The genomic inflation factor did not show any deviation (λ = 1.019).

**TABLE 1:** Main associations of the GWAS.

SNP	chr	Position	A	*r* ^2^	GWAS β	GWAS SE(β)	GWAS *p*-value	MAF (%)	MAF CEU (%)
rs114462394	3	88267399	T	0.38	3.5	0.56	2.7 × 10^−10^	5.3	10.6
rs77074982	3	88182404	G	0.39	3.3	0.53	6.7 × 10^−10^	5.9	11.1
rs567952380	9	70970401	C	0.44	3.4	0.57	1.7 × 10^−9^	5.3	6.6
rs75470026	8	4770339	C	0.55	2.5	0.43	9.4 × 10^−9^	5.2	6.1

chr: chromosome; A: effect allele, a positive beta means the effect allele favors a high PFAP; *r* ^2^: information score of imputation; GWAS β: the effect of the A allele in the GWAS, analysis; GWAS SE(β): standard error of the β; GWAS *p*-value: the p-value obtained from the GWAS; MAF: minor allele frequency of the effect allele in the 226 women of the GWAS; MAF CEU: minor allele frequency of the effect allele in 1000 genomes.

**FIGURE 2:**
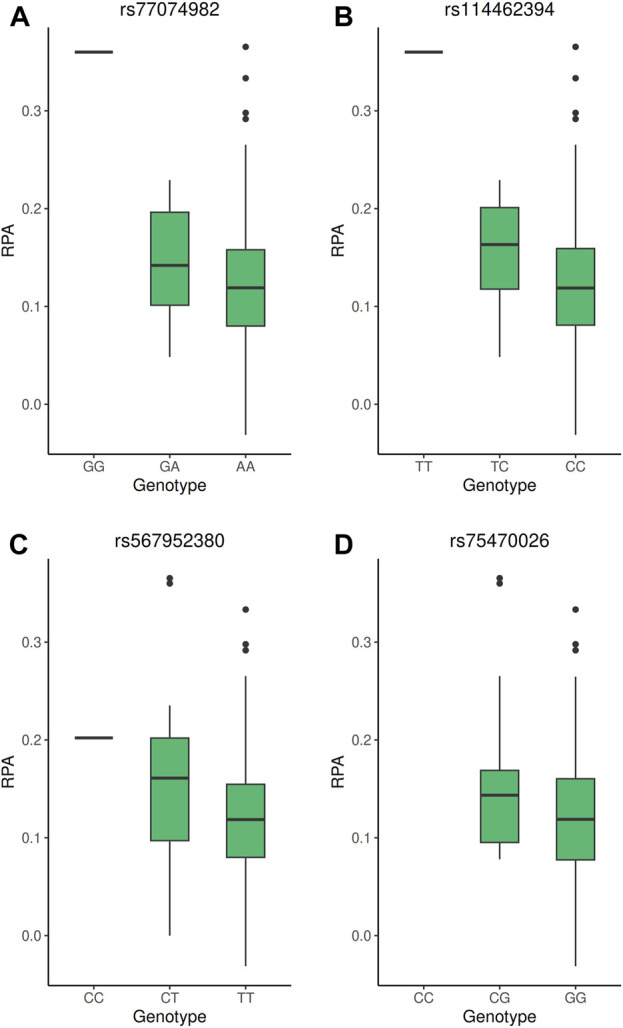
Boxplot of the PFAP according to the genotype of the GWAS significant SNP. **(A–D)** PFAP distributions according to the genotypes of the rs77074982, rs114462394, rs567952380, and rs7570026 SNP, respectively. All these SNP reached genome-wide significance threshold (5 × 10^−8^) in the GWAS under the additive model.

The strongest signal of the GWAS was detected at rs114462394 and rs77074982 SNP, which were in high LD (LD *r* ^2^ > 0.8) with three SNP (rs114203715, rs114017876 and rs114002886) and they were all located near or in the *CGGBP1* and *ZNF654* genes on chromosome 3. Among them the rs114017876 and rs77074982 SNP presented several lines of evidence for their involvement in the regulation of gene expression: eQTL effects on the *CGGBP1* gene in arterial aorta tissue and the *CHMP2B* gene in visceral adipose tissue and a high score in RegulomeDB (Table 2), (Sobin et al., 2024). The rs114017876-A allele is associated with a higher PFAP and a lower expression of *CGGBP1* while the rs77074982-G allele is associated with a higher PFAP and a higher expression of *CHMP2B* (Tables 1,2; Figures 2A,B). The second signal was carried by the rs567952380 SNP and was located on chromosome 9 in an intron of the gene *PGM5-AS1*. The rs567952380-C allele was associated with a higher PFAP in our study and with an increase of *PGM5* expression in cultured fibroblasts (Tables 1,2; Figure 2C). The last signal of the GWAS was observed for the rs75470026 SNP which was located on chromosome 8 in an intron of the *CSMD1* gene, with the C allele associated with a higher PFAP (Tables 1,2; Figure 2D).

**TABLE 2:** Results of the bioinformatics investigations.

Study	Significant SNP	SNP in LD	Gene	GWAS effect	RegulomeBD	GTEX	mQTL
GWAS	**rs114462394** **rs77074982**	**rs114462394**	*CGGBP1* (68 kb) *ZNF654* (73 kb)	T +	5		
		**rs77074982**	*CGGBP1* (intron) *ZNF654* (intron)	G +	1f	G ↗ *CHMP2B* adipose-visceral	
		rs114203715	*CGGBP1* (31 kb) *ZNF654* (36 kb)	A+	2b		
		rs114017876	*CGGBP1* (216 kb) *ZNF654* (223 kb)	A+	1f	A ↘ CGGBP1 artery-Aorta	
		rs114002886	*CGGBP1* (33 kb) *ZNF654* (41 kb)	G +	7		
	**rs567952380**	**rs567952380**	*PGM5-AS1* (intron) *PGM5* (intron)	C +	1f	C ↗ *PGM5* cultured fibroblasts	
	**rs75470026**	**rs75470026**	*CSMD1* (intron)	C +	7		
		rs78780840	*CSMD1* (intron)	T +	7		
G × E	**rs10923186**	**rs10923186**	*VANGL1* (intron)	T +	7	T ↗ *CASQ2* artery-aorta	T ↘ cg20810993
		rs10923187	*VANGL1* (intron)	T +	1f	T ↗ *CASQ2* artery-aorta T ↗ *VANGL1* nerve-Ttibial T ↗ *VANGL1* artery-Ttibial	
		rs59788048	*VANGL1* (intron)	A+	1f	A ↗ *CASQ2* artery-aorta A ↗ *VANGL1* tibial-nerve	
		rs2101025784	*VANGL1* (intron)	A+		A ↗ *CASQ2* artery-aorta A ↗ *VANGL1* tibial-nerve	
	**rs10181773**	**rs10181773**	*TRAPPC12* (intron)	A+	1f	A ↗ *TRAPPC12-AS1* skin	

The significant SNP for the GWAS or the GxE interaction analysis, appear in bold; SNP, in LD: in this column we can see the significant SNP, in bold and the SNP, in high linkage disequilibrium with them (*r* ^2^ > 0.8); GWAS, effect: “X +” mean the “X” allele favors a higher PFAP; RegulomeDB: the scoring scheme refers a higher likelihood of being functional (with 1 being higher and 7 being lower score); GTEX: “X ↗ *GENE*, tissue/cell” means the “X” allele favors a higher expression of the *“GENE*” in the” tissue/cell”; mQTL: “X ↗ CpG” means the “X” allele favors a higher methylation of the *“*CpG” site.

In the second step, only the SNP that passed the GWAS threshold of 1 × 10^−3^ (n = 7,469 SNP) were tested for an interaction with sun exposure regarding the perceived facial aging progression phenotype. In order to determine the Bonferroni threshold, we computed the LD among the 7,469 SNP to evaluate the number of independent tests (LD *r* ^2^ > 0.8). We identified 1,705 independent SNP, resulting in a threshold of 2.93 × 10^−5^. One SNP reached the Bonferroni threshold for the G × E interaction (Table 3).

**TABLE 3:** Main associations of the G × E interaction analysis.

SNP	chr	Position	A	*r* ^2^	GWAS β	GWAS SE(β)	GWAS *p*-value	Interaction β	Interaction SE(β)	Interaction *p*-value	MAF (%)	MAF CEU (%)
rs10923186	1	116227048	T	0.98	0.34	0.09	1.95 × 10^−4^	0.008	0.002	2.42 × 10^−5^	45.4	44.7
rs10181773	2	3479032	A	0.72	0.79	0.23	6.26 × 10^−4^	0.01	0.004	1.61 × 10^−4^	6.9	12.1

chr: chromosome; A: effect allele, a positive GWAS, or interaction β means the effect allele favors a higher PFAP; *r* ^2^: information score of imputation; GWAS β: the effect of the A allele in the GWAS, analysis; GWAS SE(β): standard error of the β; GWAS *p*-value: the *p*-value obtained from the GWAS; interaction β: the effect of the A allele in the interaction analysis; interaction SE(β): standard error of the β; Interaction *p*-value: the p-value obtained from the interaction analysis and correspond to the interaction only. MAF: minor allele frequency of the effect allele in the 226 women of the GWAS; MAF CEU: minor allele frequency of the effect allele in 1000 genomes.

**FIGURE 3:**
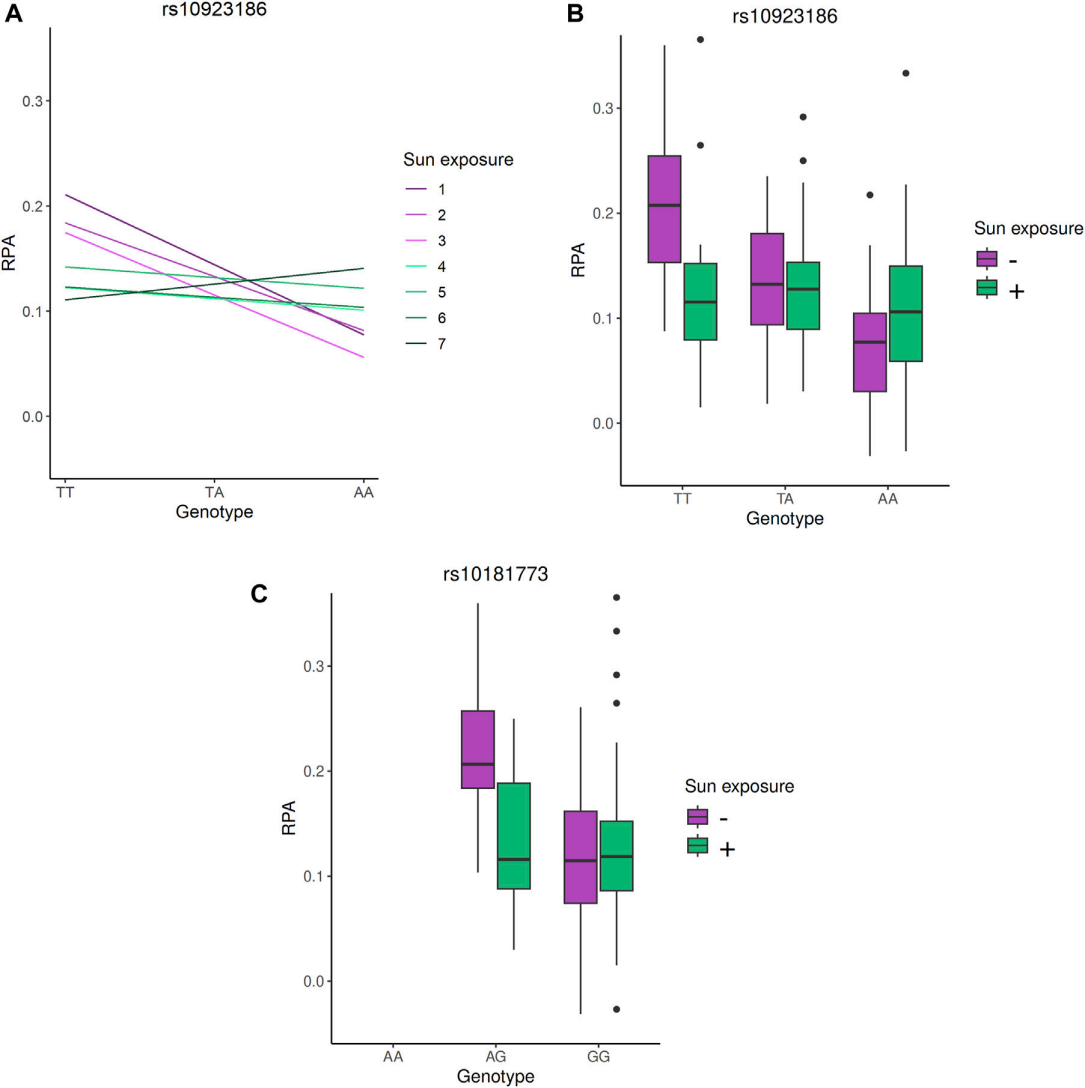
Genotype effects on PFAP in the G × E analysis. **(A)** Estimated effect of the rs10923186 T allele on PFAP within each sun exposure category. The model includes genotype, sun exposure, their interaction term, and adjustment for population structure via PC1 and PC2 and was computed with R (R Core Team, 2024). **(B)** and **(C)**: PFAP distributions according to the genotypes of the rs10923186 and rs10181773 SNP, respectively. In this figure, the seven categories of sun exposure were separated into two groups: the “-” sun exposure group includes individuals with values from 1 to 3, and the “+” sun exposure group includes individuals with values from 4 to 7. This classification into two groups was used solely for visual clarity. The rs10923186 SNP reached the Bonferroni threshold (*p*-value <2.93 × 10^−5^), while the rs10181773 SNP did not (*p*-value = 1.61 × 10^−4^).

The only significant signal involved a group of three SNP and one indel in high LD (*r* ^2^ ≃ 0.8, rs10923186, rs10923187, rs59788048 and rs111978958), with the rs10923186 SNP reaching the Bonferroni threshold (interaction *p*-value = 2.42 × 10^−5^, interaction β = 0.008, FDR = 0.04). The joint test *p*-value of the signal carried by rs10923186 was 9.23 × 10^−8^, nearly reaching the genome-wide significance threshold (5 × 10^−8^). To further characterize the interaction signal, we explored the genotype effects on the PFAP across sun exposure categories. These effects were estimated using an additive linear model including genotype, sun exposure, their interaction term, and adjusting for population stratification via PC1 and PC2 (Figure 3A) (R Core Team, 2024). We observed that the rs10923186-T allele favored a higher PFAP in the categories 1, 2 and 3 and, no effect or opposite effect in the categories 4, 5, 6 and 7 (Figure 3A). Overall, the rs10923186-T favored a higher PFAP in less exposed population (Figure 3B). The four variants (rs10923186, rs10923187, rs59788048 and rs111978958) were located on chromosome 1, near or within introns of the *VANGL1* gene. In addition to a high score for involvement in gene expression regulation (RegulomeDB), this SNP cluster was involved in an eQTL for *CASQ2* gene in artery aorta and the *VANGL1* gene in tibial nerve and in a mQTL with the cg20810993 CpG site (Table 2), (Sobin et al., 2024). In summary, through the LD analysis, we observed the rs10923186-T allele was associated with higher PFAP exclusively in the low sun-exposed population, reduced methylation at cg20810993 and higher expression of *CASQ2* and *VANGL1* genes (Figure 3A; Tables 2,3). In addition to the first signal, we obtained a trend of G × E interaction for the rs10181773 SNP, located on chromosome 2 in an intron of the *TRAPPC12* gene. The rs10181773-A allele was associated with a higher PFAP exclusively in the low sun-exposed population (FDR = 0.14, interaction *p*-value = 1.61 × 10^−4^, joint test *p*-value = 1.31 × 10^−6^; Figure 3C; Table 3). The rs10181773 SNP was involved in an eQTL with the *TRAPPC12-AS1* gene in skin notably (Table 2), (Sobin et al., 2024).

## Discussion

To explore the role of G × E interactions in facial aging, we conducted a two-step study on 226 women, focusing on perceived facial aging progression with sun exposure as the environmental factor. In the first step, we performed a GWAS to identify marginal genetic effects on PFAP and prioritize SNP for G × E interaction analysis. This initial screening identified four genome-wide significant signals and prioritized 7,464 SNP. In the second step, the 7,464 SNP were tested for interactions with sun exposure in the PFAP phenotype, resulting in one significant SNP and a second one SNP showing a trend towards association.

The strongest signal from the GWAS on the PFAP phenotype was carried by six SNP in high LD (*r* ^2^ > 0.8: rs114462394, rs77074982, rs114203715, rs114017876, rs1576144130 and rs114002886), all located on chromosome 3, near or within the *CGGBP1* and *ZNF654* genes. The *CGGBP1* gene encodes a CGG repeat-binding protein involved in DNA damage/repair, telomere metabolism, and mRNA metabolism (Singh and Westermark, 2015). *CGGBP1* has been associated with telomere protection, and given the known connection between telomere, cellular senescence, aging, and skin aging, it suggests that *CGGBP1* could play a role in facial aging via telomere protection (Singh et al., 2014; Rossiello et al., 2022; Nakamura et al., 2002). Notably, the rs114017876-A allele was associated with a higher PFAP and a lower *CGGBP1* expression, suggesting that higher *CGGBP1* expression could favor telomere protection and slow down facial aging process. The *ZNF654* gene is a protein coding gene predicted to be involved in transcriptional regulation and it is expressed broadly across tissues. It is has not yet been involved in skin aging, but it could influence multiple tissue systems relevant to PFAP, including muscles and connective tissues. The second GWAS signal was carried by the rs567952380 SNP, located on chromosome 9 within the *PGM5-AS1* gene*,* and was associated with the expression of *PGM5* in cultured fibroblasts. The *PGM5-AS1* gene codes for an antisense RNA of *PGM5* while the *PGM5* itself gene codes for a component of adherens-type cell-cell and cell-matrix junctions (Belkin et al., 1994). Besides, *PGM5* was among the top ten upregulated gene expression between progeroid versus wild-type mice and showed a downregulation of *PGM5* protein level *ex vivo* following the use of marine-derived skin care ingredients (Quintana-Torres et al., 2023; Hameury et al., 2019). Our findings were consistent with these observations, as the rs567952380-C allele was linked to increased *PGM5* expression and higher PFAP, suggesting that higher *PGM5* expression may contribute the facial aging process. The last significant signal was carried by the rs75470026 SNP located on chromosome 8, within the *CSMD1* gene. *CSMD1* encodes a large membrane-bound protein and was associated with various biological processes and conditions, including schizophrenia, the complement system, cancer, metastasis and cell migration (Akyuz and Bell, 2022). Several studies have linked *CSMD1* to aging and skin-related processes, including associations with telomere length, cognitive performance in the elderly, and hypertrophic scars (Son, Cui, and Xi, 2022; Stepanov et al., 2017; Gu et al., 2021).

For the gene and sun exposure interaction regarding PFAP, we obtained one significant signal, carried by the rs10923186 SNP, located on chromosome 1, near the *VANGL1* gene. The *VANGL1* gene encodes a protein of the tetraspanins family, which contributes to the structural organization of the plasma membrane (Singethan and Schneider-Schaulies, 2008). Interestingly, *VANGL1* was identified as a key gene for cutaneous function and was expressed during keratinization according to the Human Protein Atlas (Uhlén et al., 2015; DiTommaso et al., 2014). The keratinization process seemed relevant for skin aging, since this is the process of keratinocyte cells differentiation essential to constitute the stratum corneum, which forms the skin’s barrier against the external environment (Shetty and Gokul S 2012). In summary, our analysis showed that the rs10923186-T allele was associated with higher PFAP in the less sun exposed population, a reduced methylation level of cg20810993 CpG site and higher expression of *VANGL1*. Regarding the known impact of UV exposure on DNA methylation in the skin and the association of rs10923186 with methylation level and *VANGL1* expression level, the absence of association with PFAP in sun exposed population could be explained by UV-induced methylation changes that could lower *VANGL1* expression, thereby diminishing its effect on PFAP (Vandiver et al., 2015). Both our result and the possible role of *VANGL1* in skin, underlined the keratinization as a relevant candidate for further investigations to better understand the impact of UV exposure on facial aging. In addition to the first signal, a second SNP, rs10181773, showed a trend toward association exclusively in the low sun-exposed population. This SNP was located on chromosome 2 in the *TRAPPC12* gene was involved in eQTL effect with *TRAPPC12-AS1* gene notably in skin and skeletal muscle. *TRAPPC12* encodes a protein that is a component of the TRAPP complex, which play a role in endoplasmic reticulum to Golgi apparatus trafficking (Scrivens et al., 2011; Milev et al., 2017). It also contributes to chromosome congression, kinetochore assembly and stability (Milev et al., 2015).

The absence of association with major genes identified in previous skin aging GWAS, may reflect the difference in phenotype definitions (Ng and Chew, 2022). Most GWAS have focused on individual features of facial skin aging, such as lentigines, wrinkles, or sagging. While some have explored composite phenotypes, to the best of our knowledge, no one has ever investigated a dynamic facial aging phenotype as PFAP. The mechanisms underlying the perceived facial aging progression may involve different and complementary processes compared to those driving specific features like wrinkles. Indeed, these results may reflect the multifactorial process not limited to skin changes and involving components of face structure such as muscles, fat and ligaments (Swift et al., 2021; Cotofana et al., 2016). Several limitations could also account for these discrepancies with prior studies: the relatively small sample size of 226 individuals, the potential bias introduced by self-reported sun exposure questionnaire and the imputation uncertainty of the SNP identified in the GWAS. Sun-exposure questionnaires, however, have shown good concordance between self-reports and objective measures in several studies (Hillhouse et al., 2012; Jennings et al., 2013; Køster et al., 2017).

This study revealed four relevant genetic associations with perceived facial aging progression and one significant G × E interaction. These findings support the known link between telomere shortening/protection and aging, and suggest a potential role for keratinization in the context of facial aging and sun exposure. Although further validation in larger cohorts is necessary, these insights into the molecular etiology of PFAP through genetic approaches may help identify targeted interventions that could address not only cutaneous signs but also underlying facial structures through personalized approaches, notably in the context of a particular environment (Krutmann et al., 2021). Future studies could include functional assays targeting the identified variants (organotypic skin cultures) and integrate multi-omics data to get deeper into the understanding of the mechanisms at stake. Finally, by using a dynamic, holistic measure like PFAP, future genetic investigations may uncover novel biomarkers and mechanisms relevant for facial aging and aging.

## Data Availability

The data analyzed in this study is subject to the following licenses/restrictions: data privacy. Requests to access these datasets should be directed to Jean-François Zagury, zagury@cnam.fr.

## Abbreviations

PFAP: perceived facial aging progression.
