# Supplementary material for: Identification of gene–sun exposure interactions of GWAS-identified variants in perceived facial aging progression
Source: Front Aging. 2025 Jul 23;6:1519799. doi: 10.3389/fragi.2025.1519799 (PMC12326137; doi:10.3389/fragi.2025.1519799)

Supplementary figure S1: rate of perceived age progression

A. Composite images of photos of eight individuals in 2002. B. Composite images of photos of the same individuals in 2014.

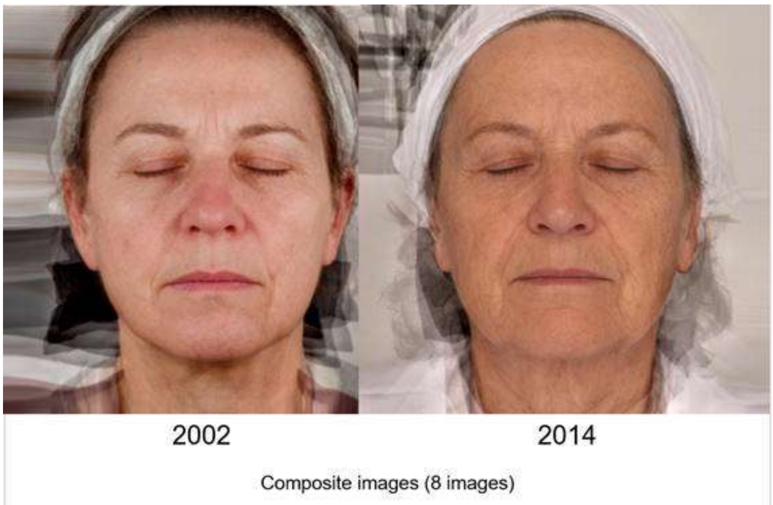

Supplementary figure S2: Principal component analysis

Scatter plot of the first two principal components (PC1 and PC2) from PCA performed on the genotypes of the 226 individuals included in the study.

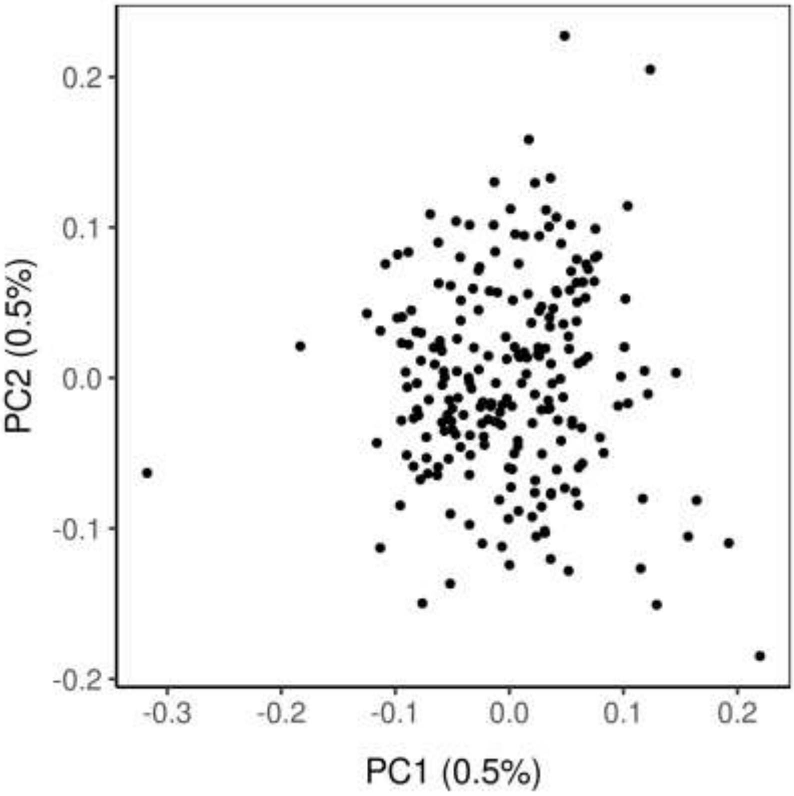

Supplement: Supplementary file 1 [file DataSheet1.pdf]
